# Autophagy and apoptosis: parent-of-origin genome-dependent mechanisms of cellular self-destruction

**DOI:** 10.1098/rsob.140027

**Published:** 2014-06-04

**Authors:** Grazyna E. Ptak, Paola Toschi, Antonella Fidanza, Marta Czernik, Federica Zacchini, Jacek A. Modlinski, Pasqualino Loi

**Affiliations:** 1 Department of Comparative Biomedical Sciences, University of Teramo, Teramo, Italy; 2 Department of Experimental Embryology, Institute of Genetics and Animal Breeding, Polish Academy of Sciences, Jastrzebiec Poland

**Keywords:** autophagy, cell death, uniparental embryo, placenta

## Abstract

Functional genomic imprinting is necessary for the transfer of maternal resources to mammalian embryos. Imprint-free embryos are unable to establish a viable placental vascular network necessary for the transfer of resources such as nutrients and oxygen. How the parental origin of inherited genes influences cellular response to resource limitation is currently not well understood. Because such limitations are initially realized by the placenta, we studied how maternal and paternal genomes influence the cellular self-destruction responses of this organ specifically. Here, we show that cellular autophagy is prevalent in androgenetic (i.e. having only a paternal genome) placentae, while apoptosis is prevalent in parthenogenetic (i.e. having only a maternal genome) placentae. Our findings indicate that the parental origin of inherited genes determines the placenta's cellular death pathway: autophagy for androgenotes and apoptosis for parthenogenotes. The difference in time of arrest between androgenotes and parthenogenotes can be attributed, at least in part, to their placentae's selective use of these two cell death pathways. We anticipate our findings to be a starting point for general studies on the parent-of-origin regulation of autophagy. Furthermore, our work opens the door to new studies on the involvement of autophagy in pathologies of pregnancy in which the restricted transfer of maternal resources is diagnosed.

## 2. Introduction

In mammals, maternal resources such as nutrients and oxygen are transferred to the embryo through a specialized organ, the placenta. The transfer of maternal resources is dependent on the correct functionality of genomic imprinting, a phenomenon that determines parental-specific gene expression [1]. Imprinted genes expressed from the paternally inherited copy increase resource transfer to the embryo, whereas maternally expressed genes reduce it [2]. Imprint-free embryos are unable to establish the correct placental vascular network. Without this network, the transfer of necessary resources is restricted, arresting the embryo's development [1,3,4]. Imprinting alterations are involved in human intrauterine growth restriction and other pathologies of pregnancy [5,6]. Knowing what strategies placentae use when faced with limited maternal resources may allow us to develop proper therapeutic measures for related placental dysfunctions.

To verify the subcellular features of placentae unable to establish contact with maternal vascularization, we used the animal model most relevant to human pregnancy studies, the sheep. The inability to establish contact with maternal vessels characterizes uniparental conceptuses, either androgenetic (AND), consisting of exclusively paternal genes, or parthenogenetic (PAR), having only maternal genes [7]. The relaxed imprinting of some genes notwithstanding, the general phenotype of AND and PAR embryos correlated with maternal- and paternal-imprint-free embryos, respectively [8]. Uni- and biparental embryos were produced *in vitro* and transferred to synchronized recipient sheep for further development using our previously described procedures [9–12].

## 3. Results and discussion

**Figure 1.**
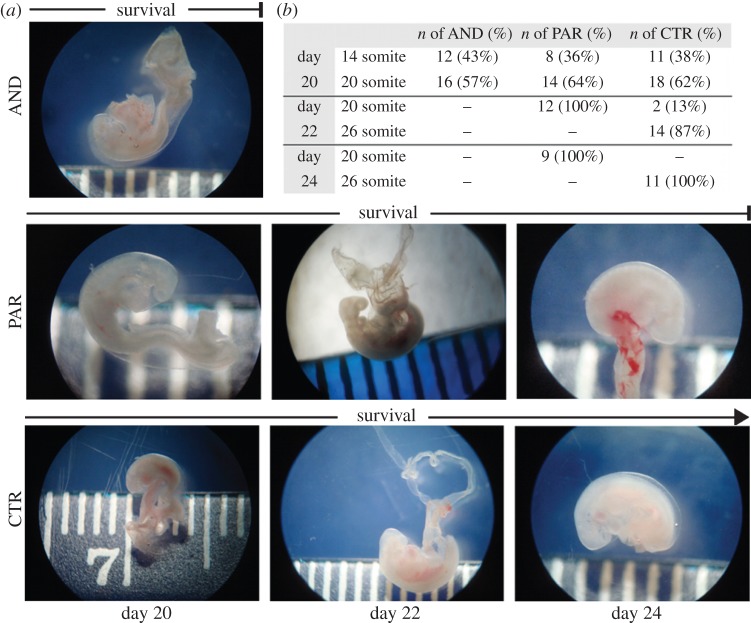
Post-implantation development of AND, PAR and CTR sheep conceptuses. (*a*) Similar size and gross morphology of AND, PAR and CTR conceptuses at day 20 of development: all had optical vesicles, two to three pharyngeal bars and closed anterior neurophores. Living (with beating heart) AND conceptuses were collected exclusively at day 20, and living, though growth-retarded, PAR conceptuses at days 22 and 24. PAR conceptuses lacked posterior limb buds and optical lens pigmentation and had reduced head size. (*b*) Most day 20 conceptuses developed to the 20 somite stage, independently of the group to which they belonged.

To determine first the extent of the growth and survival of uniparental (AND and PAR) sheep conceptuses versus bi-parental control (CTR) sheep conceptuses, these were collected at days 20, 22, 24 and 26 of development. No gross morphological differences between uni- and biparental conceptuses were noted under dissecting microscope at day 20 of development (figure 1*a*). Most of them developed to the 20 somite stage independently of their parental origin (figure 1*b*). However, there were differences in the survival of both models: AND conceptuses died prior to day 22, while PAR conceptuses died prior to day 26. PAR conceptuses isolated at days 22 and 24 were growth-retarded, as demonstrated by somite counting (figure 1*b*). On day 22 and 24 of development, the totality of PAR embryos reached the 20 somite stage, whereas the totality of biparental CTR embryos had already reached the 26 somite stage (figure 1*b*).

**Figure 2.**
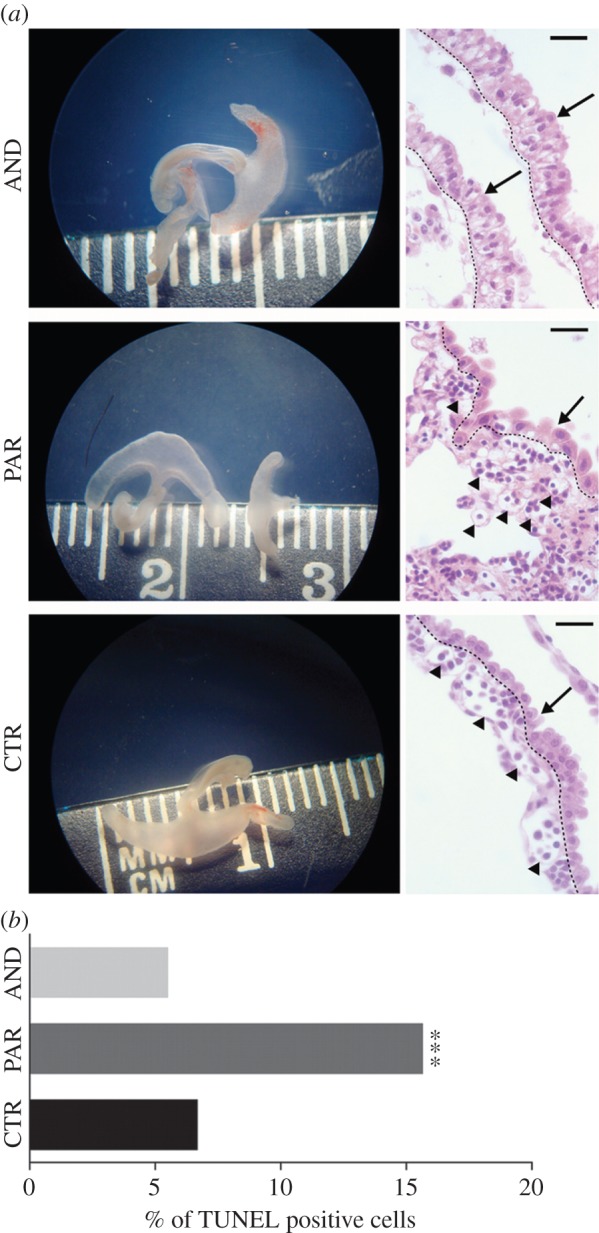
General view of AND, PAR and CTR placentae at day 20. (*a*) Similar size and gross morphology of AND, PAR and CTR placentae at day 20 of development (left column). Histological differences in development of AND, PAR and CTR placentae: a vascular network (indicated by arrowheads in the right column) was present in the allantois of PAR and CTR placentae, while completely absent in AND placentae. Trophoblastic cells of columnar shape with intense cytoplasmic vacuolization were evident in AND placentae, while PAR placentae had a low number of enlarged trophoblastic cells (trophoblastic layer indicated by arrow and delimited by dashed line). (*b*) Severe cellular death in PAR placentae revealed by TUNEL analysis of histological sections (***p < 0.001).

Furthermore, uniparental placentae at day 20 of development were similar in size (figure 2*a*) to the biparental controls. However, severe abnormalities in both uniparental models, in both the trophoblast and allantois compartments, were noted at the cellular level (figure 2*a*). The hyperproliferation of the terminally differentiated trophoblasts, together with the severely reduced vascular network in sheep AND placentae, confirms what has previously been observed in mouse [4,13]. On the other hand, the low number of trophoblast cells observed here in sheep PAR placentae were also observed in mouse PAR placentae [13,14]. As day 20 of sheep embryo development correlates with the establishment of fetal–maternal contact [15], plausibly, the subsequent death of sheep AND conceptuses and growth restriction of PAR conceptuses may be attributed to different placental defects in those models.

Imprinted genes have been shown to determine the transport capacity of the placenta by regulating its growth, morphology and nutrient abundance [16–18]. Bourc'his *et al.* [3] reported that imprint-free embryos are characterized by severe alteration of extraembryonic tissues. Indeed, uniparental embryos, carrying only either paternal (AND) or maternal (PAR) genes, cease development because of their inability to establish contact with maternal vascularization [1,19,20].

We wanted to know how paternal and maternal genomes influence cellular death in placentae that fail to establish functional connections with the maternal blood system. Maternal resource deprivation at day 20 of development did not diminish cellular viability in AND placentae while this caused massive cellular death in PAR placentae (figure 2*b*). Considering that death was more imminent for AND rather than PAR conceptuses, such a finding was surprising.

**Figure 3.**
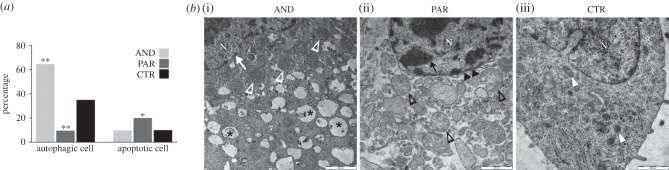
Divergent subcellular features of AND and PAR placentae at day 20 of sheep development. (*a*) Transmission electron microscopy analysis revealed that AND placentae were characterized by a very high number of autophagic cells (22/34 versus 10/28; **p = 0.0029), while PAR placentae exhibited a twofold decrease (**p = 0.0021) of autophagic cells and an increased number of apoptotic cells (8/32 versus 2/28; *p = 0.0324). (*b*) Electron micrograph of autophagic, apoptotic and normal cells from AND, PAR and biparental control placentae, respectively. (i) The AND cell had an intact nuclear membrane (white arrow), numerous autophagosomes (asterisk) and swollen mitochondria (white arrowheads). (ii) The PAR cell exhibited fragmented cytoplasm, a high number of apoptotic bodies (black empty arrowheads) and condensed chromatin (black arrow), and its nuclear membrane (black double arrowheads) lacked integrity. (iii) Control biparental cell (mitochondria: white arrowheads). N, cell nucleus. Scale bar, 1000 nm.

**Figure 4.**
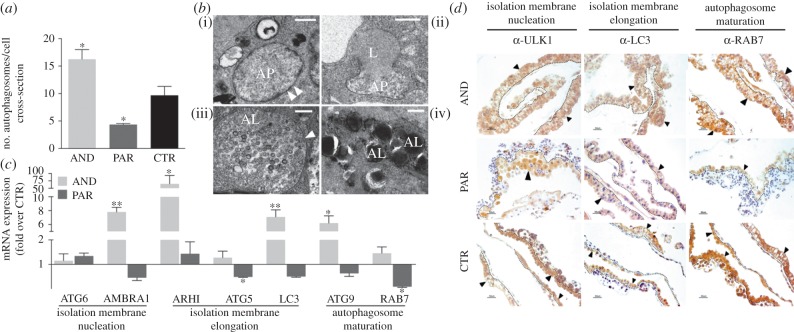
Increased level of autophagy in AND placentae. (*a*) An increased number of autophagosomes (*p < 0.05) in AND placentae and a decreased number of autophagosomes (*p < 0.05) in PAR placentae indicates the deregulation of autophagy in both uniparental models. To study whether this deregulation was due to an acceleration or blockage of autophagy at any particular stage, ultrastuctural (*b*) and molecular (*c*) analyses were performed. (*b*) The presence of all stages indicative of autophagy: (i) autophagosome—AP (white arrowheads indicate double membrane); (ii) fusion of autophagosome with lysosome—L; (iii) autophagolysosome—AL (white arrowheads indicate single vacuolar membrane); and (iv) mature autophagolysosome—AL, indicated that this process was not blocked at any particular stage in AND placentae. (*c*) Upregulated expression (more than fivefold over CTR, **p < 0.01; *p < 0.05) of genes regulating autophagy, from the initial isolation membrane nucleation and elongation to the complete autophagosome formation and maturation in AND placentae. (*d*) AND placentae also exhibited a higher protein expression of autophagosome markers (ULK1, LC3 and RAB7). Trophoblastic cells (trophoblast layer shown by arrows and delimited by dashed lines) were intensely stained in AND placentae, while less intense or absent signals of respective markers were observed in PAR placentae.

To verify how maternal and paternal genomes respond to nutritional restriction, we analysed placental ultrastructure using uniparental models. Distinct cellular morphologies of AND and PAR placentae were observed at day 20 of development (figure 3). PAR placentae had a twofold increase in the number of apoptotic cells (figure 3*a*), which is typical of the cell shrinkage and fragmentation process of the cytoplasm into apoptotic bodies. Both nuclear and cellular membranes in those cells lacked integrity and their chromatin was condensed (figure 3*b*). On the other hand, features characteristic of autophagy, such as vacuolization and an increased number of autophagosomes (i.e. double membrane vacuoles containing cytoplasmic content), swollen mitochondria together with maintained nuclear integrity, were less evident in PAR placentae (figure 3*b*). Furthermore, the expression of genes and proteins regulating autophagy was severely downregulated in PAR placentae (figure 4*c*,*d*). Conversely, severe deregulation of autophagy was noted in a majority of cells from AND placentae at day 20 of development (figure 3*a*). Autophagy or ‘self-eating’ is an intracellular degradation system, induced when cells are faced with nutritional stress. AND placental cells exhibited intense cytoplasmic vacuolization with numerous autophagosomes (figure 4*a*). Autophagosome accumulation in cell cytoplasm may reflect either the induction of the autophagic process or, alternatively, its block. One way to exclude that an increased number of autophagosomes may results from a block in the subsequent steps of autophagy is the direct estimation of the number of structures (i.e. autophagosomes and autolysosomes) involved in different steps of this process [21]. Apparently, the autophagy was increased, and not blocked, at any particular stage in AND placentae, as various stages of this process, starting from autophagosome formation through its fusion with lysosome and finally organelles digestion, were all observed with similar frequency (figure 4*b*). However, other analysis, such as LC3 turnover assay, would further strengthen our finding.

The expression of the majority of autophagy genes (figure 4*c*) and proteins (figure 4*d*) was upregulated in AND placentae. During autophagosome formation, initially a portion of cytoplasm, including organelles, is enclosed by the isolation membrane, and subsequently complete sequestration by the elongating membrane results in the formation of the mature autophagosome [22]. As demonstrated in figure 4*c*,*d*, autophagy markers involved in the different steps of autophagosome formation are deregulated.

The increased level of cellular autophagy in the placenta may protect the conceptus from nutritional stress [23]. The autophagy eliminates cellular structures and releases the breakdown products as nutrients that can be reused by the cell or exported for use by other cells [24]. Therefore, an increased autophagy in AND placentae may suggest that the paternal genome: (i) generally protects the embryo from growth restriction, and (ii) specifically protects the embryonic cells from apoptosis, as autophagy is a counterstrategy against the irreversible apoptotic signal [25]. On the other hand, the autophagy may constitute an alternative cell death pathway [22,26]. It appears that in some cellular settings, the switch between the two responses (autophagy and apoptosis) occurs in a mutually exclusive manner [27,28].

The parental contribution effect on autophagy has never before been studied. Here, we show that the AND placenta augments cellular autophagy once it is not able to transmit nutrients to the embryo, while a similar nutritional restriction will induce cell death (apoptosis) in the PAR placenta. It has been recently demonstrated that a cell whose entire genome is maternal is characterized by early cessation of cellular growth and subsequent death, while a paternal genome cell shows enhanced growth and transformation [29,30]. Downregulation of autophagy in PAR placentae is related to the involvement of paternally inherited genes in this process. At least one gene required for autophagy induction, the aplasia Ras homologue member I (*ARHI*), is known to be maternally imprinted [31,32]. Therefore, cells having diploid paternal DNA will overexpress *ARHI*, as exhaustively demonstrated in AND placentae (figure 4*c*). In turn, downregulation of apoptosis in AND placentae is related to the regulation of this type of cellular death, at least in part, by maternally inherited genes. The overexpression of the paternally imprinted gene *PHLDA2* was recently found to induce apoptosis through caspase cascade activation [33]. In our study, a *PHLDA2* transcript was not detectable in AND placentae though it was shown to be upregulated in PAR placentae (1.64-fold over CTR; *p* < 0.05). With relevance to the aberrant trophoblast layer in uniparental placentae, *PHLDA2* has also been found to affect trophoblast differentiation [34,35].

PAR placentae, which lack a paternal genome and are thus incapable of autophagy, activate an alternative cellular self-destruction pathway, that of apoptosis. AND placentae use their own cellular resources to avoid the reduction in the conceptus's growth that is observed in their autophagy-deficient counterparts, PAR placentae. However, the maximal induction of autophagy in AND placentae leads to the rapid depletion of key molecules and organelles, triggering autophagic cell death [36]. Therefore, the increased placental autophagy in AND conceptuses results in their sudden death. The gradual decrease in embryo size that accompanies the last days of development of PAR conceptuses does not occur.

The survival of sheep AND and PAR conceptuses corresponds to that in the mouse, which goes through the same developmental stages. In mouse, a specific lack of maternal imprints leads to conceptus lethality at 9.5 days post-coitum (dpc), while a lack of paternal imprints, at 13.5 dpc [1,3]. It has been recently suggested that paternal imprints may not be essential for the establishment of fetal–maternal interphase or that their effect becomes apparent later on, as paternal-imprint-free embryos survive longer than their maternal-imprint-free counterparts [1]. Besides the differences between maternal- and paternal-imprint-free placental development, our study suggests that the different timing of embryonic death in maternal- and paternal-imprint-free embryos is, at least in part, due to divergent cellular death strategies.

In the light of our findings, it appears that the maternal genome drives the development of the embryo only when an adequate transfer of resources is guaranteed. By contrast, the paternal genome seeks the survival of the conceptus ‘at all costs’, and therefore uses autophagy as a rescue strategy. Such an interpretation is in agreement with the imprinting hypothesis about excessive utilization of available resources directed by the paternal genome and the protection against it from the maternal counterpart [37].

Studies on placental cell death during pregnancy complications such as intrauterine growth restriction and pre-eclampsia have thus far focused exclusively on apoptosis [38]. However, as we have shown, autophagy is also involved as a mechanism in placental cell destruction. As imprinting disorders are involved in the aetiology of these pregnancy complications, understanding the underlying control of autophagy in pathological placentae, in addition to apoptosis, is necessary to paint the complete picture of how placental cell death leads to conceptus growth aberrancies.

## 4. Material and methods

### 4.1. Embryo production

*In vitro* embryo production methods were adapted from those previously described [12,39]. Briefly, ovaries were obtained from the abattoir. Collected oocytes were matured in bicarbonate-buffered TCM-199, supplemented with 2 mM l-glutamine, 100 mM cysteamine, 0.3 mM sodium pyruvate, 5 g ml^−1^ FSH (Ovagen, ICP, Auckland, New Zealand), 5 g ml^−1^ LH, 1 g ml^−1^ 17-β-oestradiol and 10% fetal bovine serum under 5% CO_2_ at 38.5°C for 24 h. For parthenogenetic embryo production, *in vitro* matured oocytes were activated with a combined treatment of ionomycin and 6-dimethylaminopurine, in synthetic oviductal fluid (SOF) medium, as previously described [9]. For androgenetic embryo production, the embryos were produced by *in vitro* fertilization of enucleated metaphase II oocytes as previously described [10]. For *in vitro* fertilized embryo production, partially denuded matured oocytes were transferred into 50 µl drops of bicarbonate-buffered SOF enriched with 2% (v : v) heat-inactivated estrous sheep serum, 2.9 mM calcium lactate and 16 mM isoproterenol. Fertilization with frozen–thawed ram semen was carried out at a final concentration of 5 × 10^6^ sperm ml^−1^, and fertilized eggs were kept in 5% CO_2_ at 38.5°C overnight. For embryo culture, all classes of embryos were transferred into 20 µl drops of SOF enriched with 1% (v : v) Basal Medium Eagle essential amino acids, 1% (v : v) Minimum Essential Medium, non-essential amino acids (Gibco), 1 mM glutamine and 8 mg ml^−1^ fatty-acid-free BSA (SOFaa-BSA). Embryos were cultured in a humidified atmosphere of 5% CO_2_, 7% O_2_, 88% N_2_ at 38.5°C, and the medium changed at day 3 and day 5.

### 4.2. Embryo transfer and recovery samples

All animal experiments were performed in accordance with DPR 27/1/1992 (Italian Animal Protection Regulation) and conformed to the European Community regulation 86/609. Embryo transfer (ET) and sample recovery were carried out as previously described [12]. Placentae (chorionallantoid tissues) from live fetuses were snap-frozen in liquid nitrogen or fixed for subsequent analysis. A total of 56 CTR, 28 AND and 43 PAR fetuses and their placentae were recovered from 50 Sardinian sheep.

### 4.3. Gene expression analysis

Expression analysis was carried out as previously described [12]. Total RNA integrity was assessed by a 2100 Bioanalyzer (Agilent Technologies, Waldbronn, Germany). Samples with an RNA integrity number of at least 8.5 were reverse-transcribed and used for gene expression analysis with specific primer pairs (LC3 (JQ035664.1) FW: 5′-atggtatacgcctctcagg-3′, RV: 5′-ttcccaaagctgaatgtgc-3′; ATG5 (JQ035661.1) FW: 5′-aaggaccttctacactgtcc-3′, RV: 5′-atctgtagacacaggtcg-3′; ATG9 (JQ035663.1) FW: 5′-aagacgtgctggctgtgg-3′, RV: 5′-ttgtactggaagagctgg-3′; ATG6 (JQ035662.1) FW: 5′-ttgaaactcgccaggatgg-3′, RV: 5′-ttgagctgagtgtccagc-3′; AMBRA1 (NM_001034522) FW: 5′-atctgggatttacacggtgg-3′, RV: 5′-ttgctgtgagtaagtagtgtcc-3′; ARHI (NM_001127282.1) FW: 5′-gaaggaaggtgctgcctatg-3′, RV: 5′-gtcttggggatctgggattt-3′; RAB7 (NM_001035081.1) FW: 5′-ctgacgaaggaggtgatggt-3′, RV: 5′-aagggtcttgaacgtgttgg-3′; β-actin (NM_001009784) FW: 5′-aatcgtccgtgacatcaag-3′, RV: 5′-ttcatgatggaattgaagg-3′).

### 4.4. Immunohistochemistry and TUNEL assay

Twenty-day placental tissues were fixed overnight in 4% paraformaldehyde, and 5 µm sections were used for immunohistochemistry and TUNEL assay. Different primary antibodies were incubated: LC-3, 4 μg ml^−1^ (ab58610); ULK-1, 1 : 1000 (ab65056); and Rab-7, 10 μg ml^−1^ (ab77993) (all from Abcam, Cambridge, UK). Antibody binding was visualized using Universal LSAB (Dako, Glostrup, Denmark). Also, 5 µm sections were TUNEL assayed using ApopTag fluorescein kit (Millipore, MA, USA) according to the manufacturer's protocol. Immunohistochemistry images were captured using the Nikon Eclipse E600 light microscope (Nikon, Melville, NY, USA), while TUNEL assay images were obtained using confocal microscope Laser Sharp 2000 (Biorad, Milan, Italy).

### 4.5. Transmission electron microscopy

Placental tissues were fixed in 2.5% glutaraldehyde for 24 h and postfixed in 2% OsO_4_ for 4 h. Samples were dehydrated through a graded series of ethanol and infiltrated with Epon resin in 100% acetone, infused twice for 1 h in pure epon resin and polymerized for 24 h at 65°C; 60 nm sections were examined on a LEO 912AB electron microscope (Leo Electron, Thornwood, NY, USA). Images were captured by the Slow Scan CCD (Proscane) using EsiVision Pro v. 3.2 software (Soft Imaging Systems GmbH).

### 4.6. Statistical analysis

Statistical analysis was performed using Instat 5 (GraphPad Software for Science, San Diego, CA, USA). Data reported are the mean ± s.e.m. and were analysed using the non-parametric Mann–Whitney *t-*test (figure 4*a*,*c*). Data expressed as percentages were analysed using the Fisher's exact test (figure 3*a*) or *χ*^2^-test (figure 2*b*). Only *p*-values < 0.05 were considered significant.
